# Small area estimation of child undernutrition in Ethiopian woredas

**DOI:** 10.1371/journal.pone.0175445

**Published:** 2017-04-14

**Authors:** Thomas Pave Sohnesen, Alemayehu Azeze Ambel, Peter Fisker, Colin Andrews, Qaiser Khan

**Affiliations:** 1 Independent Consultant, Copenhagen, Denmark; 2 World Bank, Washington DC, United States of America; 3 Department of Food and Resource Economics, Changing Disasters, University of Copenhagen, Copenhagen, Denmark; Kenya Medical Research Institute - Wellcome Trust Research Programme, KENYA

## Abstract

Reducing child undernutrition is a key social policy objective of the Ethiopian government. Despite substantial reduction over the last decade and a half, child undernutrition is still high; with 48 percent of children either stunted, underweight or wasted, undernutrition remains an important child health challenge. The existing literature highlights that targeting of efforts to reduce undernutrition in Ethiopia is inefficient, in part due to lack of data and updated information. This paper remedies some of this shortfall by estimating levels of stunting and underweight in each woreda for 2014. The estimates are small area estimations based on the 2014 Demographic and Health Survey and the latest population census. It is shown that small area estimations are powerful predictors of undernutrition, even compared to household characteristics, such as wealth and education, and hence a valuable targeting metric. The results show large variations in share of children undernourished within each region, more than between regions. The results also show that the locations with larger challenges depend on the chosen undernutrition statistic, as the share, number and concentration of undernourished children point to vastly different locations. There is also limited correlation between share of children underweight and stunted across woredas, indicating that different locations face different challenges.

## Introduction

Child undernutrition is an important public health problem in developing countries, as reflected by undernutrition being rated as the first priority among the world’s ten most important challenges by the Copenhagen Consensus[1]. Its effect could be either immediate through increased child morbidity and mortality or later in adult life by affecting health and labor market outcomes. Despite considerable progress in reducing undernutrition—underweight (too low weight for age) fell from 41 to 25 percent of children, stunting (too short for age) fell from 58 to 40 percent of children, and wasting (too low weight for height) fell from 12 to 9 percent of children from 2000 to 2014[2]—Ethiopia is still among the countries in the world with highest child undernutrition prevalence. Taken together, about 48 percent under the age of five were undernourished (being either stunted, underweight, or wasted) in 2014, equivalent to approximately 6.3 million children[2].

The government is aware of this challenge and has set out to reduce stunting to 30 percent and wasting to 3 percent by 2015[3]. Programs to address these challenges include: increasing agricultural productivity, promoting girls’ education, immunization, integrated management of neonatal and childhood illnesses, improved access to water and sanitation, family planning, prevention of mother-to-child transmission of HIV, skilled birth delivery, and delaying of pregnancy[3].

The prevalence of different types of undernutrition differs by location in Ethiopia, indicating that different locations face different challenges[4]. International evidence also shows that many different interventions improve undernutrition outcomes, but also that similar interventions have different impacts in different settings and locations[5]. Various interventions and correlations have also been identified in Ethiopia to matter for undernutrition outcomes, including: shocks and food aid[6, 7], maternal education and nutritional status[8–15], ownership of selected assets such as cows[16], food availability and diversity[17], access to trained and educated health professionals[18], access to safe water and feeding practices[19, 20].

Rajkumar et al.[4] evaluate targeting of efforts to reduce undernutrition in Ethiopia and conclude, among other things, that:

1. Most nutrition-related programs focus exclusively on a subset of the country’s woredas and these are difficult or impossible to identify accurately because of a poor nutrition information system.
2. Many programs use food insecurity as a proxy for nutrition insecurity, even though these are not highly correlated. Further, woreda targeting for food insecurity is often based on dated information.

They conclude: “*Ethiopia’s malnutrition rate could probably be much reduced by shifting some of the programs from the woredas with a high concentration of major programs into woredas with high malnutrition*”. This paper addresses this shortage of information by estimating levels of stunting and underweight for all woredas.

As in many other developing countries, there is large urban-rural divide in prevalence in Ethiopia. Child stunting is 16 percentage points lower in urban (27.0%) than rural (42.6%) areas.[8] The urban-rural divide is large, but there are also notable variations, particularly in underweight, across regions[4]. The importance of the spatial dimension has been observed in Ethiopia[15] and in other countries, and the result is often strong even after controlling for other correlates[21]. Undernutrition regressions for Vietnam, South Africa, Pakistan, and Morocco suggest that community-level effects are of great importance[22]. Fuji[22] for instance, in his undernutrition regressions for Cambodia finds that individual-level and household-level variables explained 20 to 30 percent of the variation in the z-score, while he was able to increase the explanatory power of the model to about 40 to 60 percent by including geographic variables and interaction terms. Alderman and Christiaensen[9] is an exception, as they find in their analysis of Ethiopia that location does not matter significantly once they control for other correlates.

The local estimates of undernutrition in this paper is limited to underweight and stunting as an imputation model with reasonable accuracy was not found for wasting. In OLS regressions between z-scores for wasting and household characteristics, it was not possible to find explanatory variables that explained the z-score. A weaker correlation between household characteristics and z-scores for wasting is often observed, and previous country studies using small area estimation (henceforth SAE) for undernutrition in Bangladesh[23], Cambodia[22, 24], Ecuador, Panama, Dominican Republic[25] and Tanzania[26], were also unable to find a suitable model for wasting. Other undernutrition SAE country studies include stunting in Brazil[27]; and stunting, underweight and wasting in Nepal[28]. The ability to model wasting depends both on the range of variables available in the census, and on the correlation between z-scores for wasting and household characteristics in each country.

This paper contributes in two aspects. First, it addresses the shortage of updated evidence on local undernutrition prevalence by estimating undernutrition prevalence for all woredas in 2014, using SAE. These estimates are the first for Ethiopia. It hereby adds to the rich literature on *who* is undernourished by showing *where* to find the undernourished. Second, an evaluation of the SAE models shows that geographical targeting using woreda levels of undernutrition is a viable strategy, as the SAE models have as good or better predictive power than common known correlates of undernutrition. The SAE estimates show that different undernutrition statistics point to different woredas having the largest problems. The different statistics, combined with the correlation between them, could be used to select the most appropriate type of intervention in different locations.

The rest of the paper is organized as follows: Section 2 describes the data, Section 3 presents the SAE methodology, Section 4 shows results, while Section 5 concludes.

## Data

The primary data source of undernutrition in this study is the Ethiopia Mini Demographic and Health Survey (EMDHS) 2014[2]. The EMDHS 2014 is a stratified nationally representative survey, including 5,579 children under five years of age. The survey follows the Demographic Health Surveys[29] standard survey design, although some sections, such as HIV and immunization, were not included. Detailed information about the sampling is available in the survey’s report[2].

Undernutrition is measured as share of children moderately or severely undernourished (below two standard deviations from the mean) based on z-scores for stunting (height for age), and underweight (weight for age). Z-scores were calculated using EMDHS and the 2006 WHO growth standards[30].

To obtain estimates of undernutrition prevalence at woreda levels, EMDHS is combined, through SAE, with the 2007 Census. The 2007 census has two formats—a long and a short format. The long format is richer in terms of data and in addition to demographic information, it includes information on assets, housing characteristics, education, fertility, and mortality. A randomly selected 20 percent of the households received the long form, while the other 80 percent received the short form that only covered basic demographics. In addition to the census the agricultural zones and quality of soil[31] is added as explanatory variables.

The EMDHS and a 10 percent sample of the 2007 census is available at request from the Ethiopian Central Statistical Agency[32].

## Small area estimation methodology

Woreda level estimates of undernutrition are estimated by SAE. The basic idea is to construct a prediction model between the z-score and observable household and location characteristics in EMDHS. This model is then used to predict a z-score for every child using the census. This paper relies on the SAE method developed in Elbers, Lanjouw, and Lanjouw[33] (henceforth ELL). This specific method is attractive, as it can be easily implemented in the freeware program PovMap2[34]. There are numerous technical variations of the SAE methodology. For recent overview of variations in methodology and application of SAE see Rao et al. [35] and Pratesi [36].

The methodology relies on three steps. In step one, a set of explanatory variables which are similarly defined and distributed in the survey and the census are identified. Restricting the explanatory variables (X below) to those that are time invariant, ensures that the model estimates z-scores for 2014 (the year of the survey) and not 2007 (the year of the census). The section below, provides more details on step one.

In step two, a z-score model is constructed in the survey data:

$$z_{i}=X_{ih}^{survey}\beta +Z^{\prime }\gamma +u_{ih}$$ (1)

where $X_{ih}{}^{\prime }$ is the vector of explanatory variables for child *i* in household *h*, *β* is the vector of coefficients, *Z*′ is the vector of location specific variables, *γ* is the vector of coefficients, and *u*_*ih*_ is the error term due to the discrepancy between the imputed z-score and the actual value. $X_{ih}{}^{\prime }$ is child and household level variables that have similar definitions and distributions in survey and census, while *Z*′ includes location-specific averages or other transformations of variables found in the census that cannot be found in the survey, and external variables, such as geo-spatial variables that can be added to both the survey and the census.

In step three, undernutrition estimates and their standard errors are computed via simulations using the estimation model. There are two sources of errors involved in the estimation process: errors in the estimated regression coefficients ($\hat{\beta }$, $\hat{\gamma }$) and the disturbance terms, both of which affect undernutrition estimates and the level of their accuracy. In ELL, a simulated z-score is calculated for each census child by a predicted z-score as in (2):

$$\hat{z_{i}}=\;X_{ih}^{survey}\hat{\beta }+Z^{\prime }\hat{\gamma }+\hat{u_{ih}}$$ (2)

These simulations are repeated a large number of times (100 in the case of this application), with new values for *β*, *γ*, and *u*_*ih*_ drawn from their distributions, in each repetition. The distributions of *β*, *γ* are obtained through GLS, while the distribution of *u*_*ih*_ is obtained through a non-parametric approximation following Elbers and van der Weide[37]. All the estimation is automated within the PovMap2 program.

For any given location (such as a woreda), each simulation is used to calculate the share of children underweight or stunted (a z-score below negative two standard deviations). The mean across the simulations of an undernutrition statistic provides a point estimate of the statistic, and the standard deviation provides an estimate of the standard error.

In most applications of ELL, the error term *u*_*ih*_ is decomposed into two independent components: *u*_*ih*_ = *η*_*c*_ + *ε*_*ih*_, where *η*_*c*_ is a cluster-specific effect and *ε*_*ih*_ an individual effect. In this application, the error term has not been split into a cluster and individual effect, as no cluster effect was found. The lack of a cluster effect is likely closely related to the sampling design of EMDHS, as it is designed with few EAs within each region and relatively large samples within each EA. A cluster effect would often be based on EAs, and with limited number of EAs within each region, it was not possible to establish a distribution of cluster effects.

## Results

Results are presented in three sections. Section one documents the application of the SAE methodology to Ethiopian data and evaluate the quality of the application, section two evaluates if SAE models, with the limited data are powerful predictors compared to other more common correlates of undernutrition, while section three illustrates the spatial patterns in undernutrition across Ethiopian woredas.

### Application of SAE to Ethiopian data

SAE estimates of stunting and underweight is done at woreda level. Woreda is the third administration layer after region and zone. Each woreda is also divided into kebeles, which are the smallest administrative units in Ethiopia. A woreda on average has around 12000 children, with a standard deviation of 8000, with around 2000 children at the 5 percentile and 28500 children at the 95 percentile.

The SAE estimates are based on regional z-score models. Regional models have the advantage of fitting data relevant to local circumstances, although they limit the number of variables in each model as the number of observations decreases. The EMDHS 2014 survey sampled all woredas in the city regions of Harari, Dire Dawa, and Addis Ababa, and the survey estimates from EMDHS were used for those three regions as opposed to SAE estimates.

The Ethiopian census has both a short and a long form, and to increase model fit, the models use the long form, including enumeration area (EA) averages of all variables from the census whether found in the survey or not. Separate models for the long and short form are theoretically possible. Unfortunately, this is not possible using the PovMap software, as the program cannot handle the likely correlation between two such models, which would result in inaccurate estimates of undernutrition standard errors.

To mitigate issues arising from the time interval between the 2007 Population Census and EMDHS 2014, only variables whose distributions are time invariant between the two are used in the z-score models. The PovMap program jointly shows the distributions from each data source for each variable, allowing a visual inspection of the similarity of the distributions. PovMap also provides a frequency chi-square test for categorical variables and a Kolmogorov-Smirnov test for continuous variables. These tools are used to judge similarity of the distributions and if they are time invariant or not. A strict statistical cut off has not been applied as there is a tradeoff between model contribution and similarity of variables. Further, in some cases a categorical variable might seem perfectly similar, but one category when used as dummy might not pass the statistical threshold though the overall categorical variable does. In such a case, the one category dummy would be allowed to enter the model despite not passing the statistical test on its own. To avoid overfitting skewed dummy variables with a mean smaller than 0.05 in the survey are also excluded from the models. Variable selection into the estimation models was based on contribution to adjusted r-square, and correlation between variables (highly correlated variables were excluded). Further, all models include variables generated at levels above the household level, as too high spatial correlation in the error term relative to the overall error term can jeopardize accuracy of estimated standard errors of undernutrition indicators[38, 39]. The models also include variables as number of children in household and children close in age, which capture within household correlation. See also Jones and Haslett[28], as well as Fujii[22], for alternative ad hoc modeling solutions for within household correlation. S1 Table shows the survey and census means for all variables included in the final models. All models include variables that are based on EA or higher-level census means; these are not included in S1 Table, as they are comparable by definition.

The models have adjusted r-squares similar to those of other undernutrition regressions, particular those used for undernutrition maps (0.06 to 0.27 for stunting and between 0.08 and 0.14 for underweight). Some studies have succeeded in developing undernutrition models for SAEs of undernutrition with R-squares as high as 0.6 and 0.7[22]. However, some express concern for overfitting with R-squares above 0.35[27]. The full regional regression models are enclosed in S2 Table.

As a check on model accuracy, Table 1 shows the model-imputed undernutrition prevalence and those measured in EMDHS at regional level. The table shows that measured undernutrition prevalence in EMDHS and the estimated prevalence from census are similar, and none of the estimates are outside the 95 percent confidence interval of the measured levels.

**Table 1:** Measured and imputed undernutrition prevalence, 2014.

Region	Imputed	Survey	95% confidence interval for survey estimates		Observations in survey
	undernutrition rate	undernutrition rate			
	Stunting				
Tigray	0.440	0.459	0.380	0.537	435
Afar	0.404	0.465	0.396	0.533	565
Amhara	0.413	0.432	0.376	0.488	518
Oromia	0.41	0.385	0.334	0.435	635
Somali	0.42	0.36	0.292	0.427	565
B. Gumuz	0.412	0.406	0.353	0.459	426
SNNP	0.421	0.443	0.391	0.496	673
Gambela	0.275	0.219	0.149	0.288	403
	Underweight				
Tigray	0.351	0.312	0.262	0.364	445
Afar	0.496	0.455	0.408	0.514	608
Amhara	0.341	0.291	0.248	0.343	523
Oromia	0.271	0.229	0.196	0.286	656
Somali	0.403	0.386	0.331	0.46	591
B. Gumuz	0.327	0.282	0.212	0.364	438
SNNP	0.289	0.260	0.213	0.325	699
Gambela	0.224	0.199	0.126	0.273	412

Source: DHS 2014 and imputed values from models.

**Fig 1:**
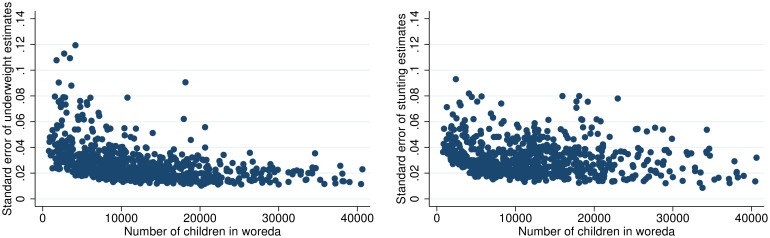
Standard errors of underweight and stunting prevalence, and number of children in woredas.

As also illustrated in Table 1, both measures from surveys and the estimates from models come with standard errors. The estimated standard errors of undernutrition levels across woredas are on average similar and a little larger than those observed at regional levels from the survey. Woredas with smaller populations of children generally come with larger standard errors. Fig 1 illustrates the standard errors in relation to the number of children in each woreda. It illustrates both how woredas with lower number of children have larger standard errors (the left tail), and how standard errors are larger for stunting than underweight. This is so despite stunting models having higher r-square and is due to larger underlying variation in z-scores for stunting than for underweight. As example of the influence of standard errors: Sixty-five percent of woredas have underweight levels above the national average of 26.6, while only 41 percent of these are significantly above the national average (based on the woreda estimates lower bound of the 95 percent confidence interval being above the national mean). Similarly, 46 percent of woredas have stunting levels that are above the national average, while only 14 percent are significantly above that level (again using the 95 percent confidence interval).

### Are SAE estimates of undernutrition in woredas a useful targeting mechanism?

As highlighted in the introduction, existing literature points to weaknesses in data for geographical targeting, but can models utilizing only a reduced set of explanatory variables (as the set of potential variables is reduced to those available in the census that are time invariant, see above) really predict undernutrition? Most variables highlighted in the literature and described in the introduction are not available in the census, and hence cannot be used in our predictive models. Given available data, a complete analysis of this question is not possible, but it is possible to compare the predictive power of the SAE models with more traditional correlates of undernutrition found in the EMDHS survey. The literature on correlates of malnutrition include both variables that can be linked directly to causality and some with more indirect links to causality. For SAE models the focus in on predictive power and causality is not an requirement. To compare the SAE models predictive power to other more common correlates of undernutrition, an OLS regression of z-scores is estimated in the EMDHS with both common undernutrition correlates and the SAE models. The regional SAE models are included in the national regression by multiplying variables in each model by regional dummies. In the interest of space, the SAE models are not shown in Table 2, but the complete models can be seen in S2 Table. The regressions further include the following common correlates of undernutrition: a) access to health services approximated by households utilizing any pre- or post-natal services; b) access to clean water approximated through households utilizing protected water source; c) lack of access to toilet facilities; d) access to food and other necessities, and or knowledge approximated by wealth measured by the EMDHS asset index; e)mothers’ education; f)children’s age; and g)regional dummies. Among these common correlates of undernutrition, only the children’s age (at individual level) and mothers’ education (at an aggregated level) are found in the census, and therefore among the potential variables in the SAE models.

**Table 2:** OLS regression of z-scores.

Column	1	2	3	4	5	6	7	8
Dependent variable: z-score	HAZ	HAZ	HAZ	HAZ	WAZ	WAZ	WAZ	WAZ
HH has access to protected water		0.03	-0.06	0.07		0.02	-0.03	0.05
		(0.11)	(0.12)	(0.10)		(0.07)	(0.08)	(0.07)
HH has no access to toilet		-0.10	-0.02	-0.06		-0.01	0.04	0.01
		(0.10)	(0.10)	(0.08)		(0.07)	(0.06)	(0.06)
HH utilize health service		0.05	0.05	0.05		0.06	0.05	0.05
		(0.10)	(0.09)	(0.09)		(0.07)	(0.07)	(0.07)
Mothers’ education (no education is the excluded category)								
Primary		0.21**	0.07	0.14		0.19**	0.13*	0.16**
		(0.09)	(0.10)	(0.09)		(0.07)	(0.08)	(0.07)
Secondary		0.73***	0.59**	0.45		0.72***	0.65***	0.58***
		(0.25)	(0.27)	(0.28)		(0.15)	(0.16)	(0.15)
Tertiary		1.42***	0.71**	0.68**		1.11***	0.75**	0.81**
		(0.29)	(0.29)	(0.29)		(0.31)	(0.33)	(0.32)
Wealth Index			0.00**	0.00			0.00*	0.00
			(0.00)	(0.00)			(0.00)	(0.00)
Age of child (age 4 excluded category)								
Less than 1			1.70***	1.45***			0.94***	0.46**
			(0.14)	(0.27)			(0.09)	(0.19)
1 through 2			0.12	-0.21			0.17**	0.06
			(0.13)	(0.24)			(0.08)	(0.14)
2 through 3			-0.34***	-0.35**			0.03	0.02
			(0.12)	(0.17)			(0.09)	(0.13)
3 through 4			-0.25**	-0.26			0.08	-0.03
			(0.10)	(0.16)			(0.07)	(0.13)
Regional dummies	x	x	x	x	x	x	x	x
**SAE models**	x			x	x			x
Constant	0.45	-1.07***	-1.02***	-2.25***	-0.37	-1.21***	-1.31***	-1.31***
	(0.68)	(0.20)	(0.23)	(0.42)	(0.34)	(0.13)	(0.14)	(0.38)
Number of observations	3,838	3,838	3,838	3,838	3,976	3,976	3,976	3,976
R2	0.241	0.023	0.176	0.247	0.152	0.040	0.107	0.164

The asterisks indicate the significance level:

*** p<0.01,

** p<0.05,

* p<0.1.

Standard errors in parentheses. Regressions take sample design and household weights into account by using Stata’s svy command. The city regions of Harari, Dire Dawa, and Addis Ababa are excluded as there are no SAE model for those locations. Data: EMDHS 2014.

All commonly correlated variables for both stunting and underweight have the expected signs, though some of the insignificant variables change sign when controlling for wealth and children’s age (column 2–4 and 6–8, Table 2). Similar patterns have been observed in Ethiopia before, based on other data sets[40].

The common correlates/indicators of undernutrition (column 2–4 and 6–7) have r-squares of 0.18 and 0.11 for stunting and underweight respectively, with most of the explanatory power coming from the wealth index and children’s age. In comparison, the SAE models (column 1 and 5 in Table 2) have r-squares of 0.24 and 0.15 for stunting and underweight. Hence, the SAE models have more about 50 percent more predictive power then “common undernutrition correlates”. Evaluated jointly, the traditional correlates do add predictive power to the SAE models, but it is not a large contribution (column 4 and 8, Table 2).

### Spatial patterns in woreda estimates of undernutrition

**Fig 2:**
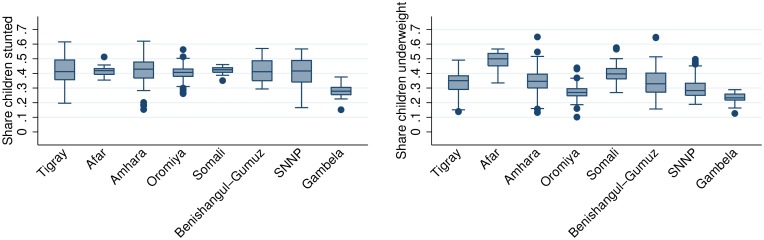
Variation in share of children stunted and underweight across woredas in each regions. The bar in center of box is the median stunted and underweight prevalence across woredas in each region. The box is the 25th and 75 percentile; dots are outside values.

A first observation, based on the SAE-obtained woreda level estimates, is that only a few regions are homogenous with similar levels of undernutrition across woredas (Fig 2). This is illustrated in Fig 2 by the wide ranges of levels across woredas within each region. In almost all regions, woredas with higher levels have more than twice the level of those with lower levels. The variation in number of woredas in each region influences the graph, though it does not seem to drive results as both Gambella, with only 13 woredas, and Oromia, with 277 woredas, both have relatively small variation. The variation in undernutrition across woredas within regions indicates that there is scope for spatial targeting within most regions.

**Fig 3:**
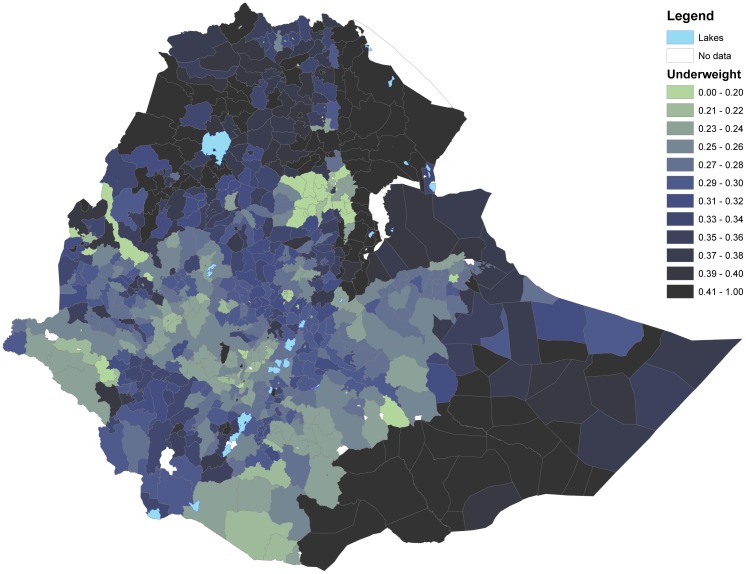
Share of children underweight in each woreda. The figure shows share of children underweight.

**Fig 4:**
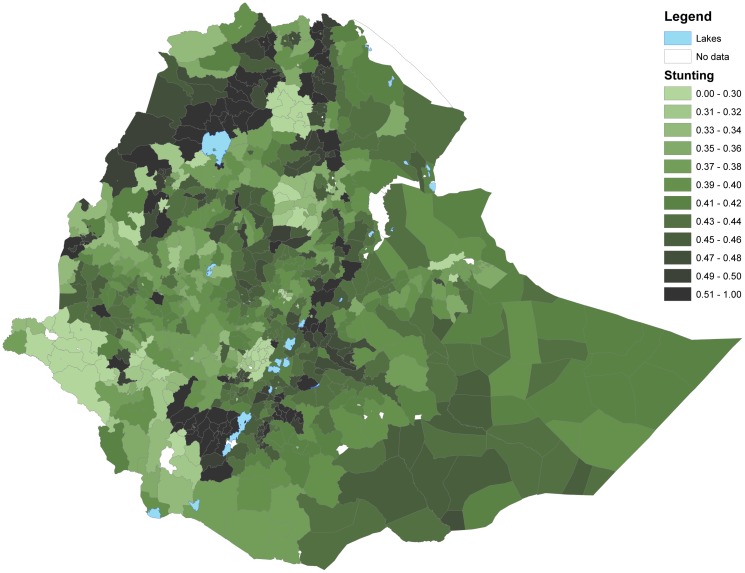
Share of children stunted in each woreda. The figure shows share of children stunted.

**Fig 5:**
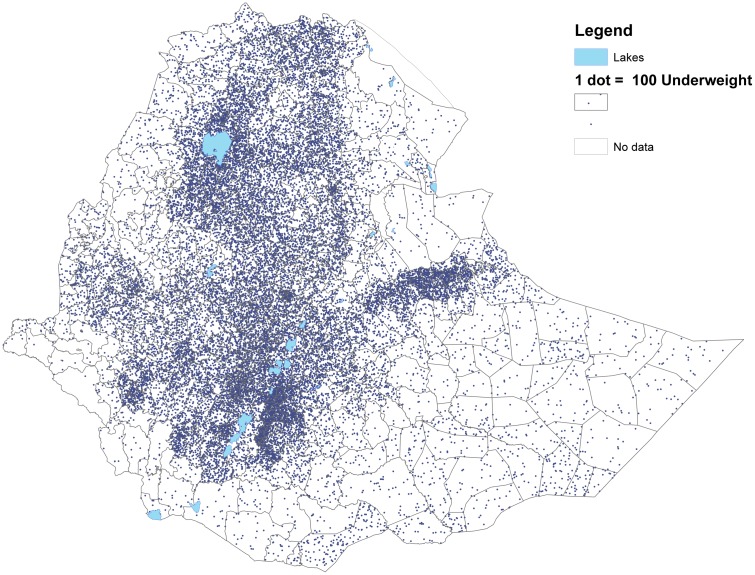
Number of children underweight in each woreda. The figure shows the number of underweight children. Each dot represent 100 underweight children.

**Fig 6:**
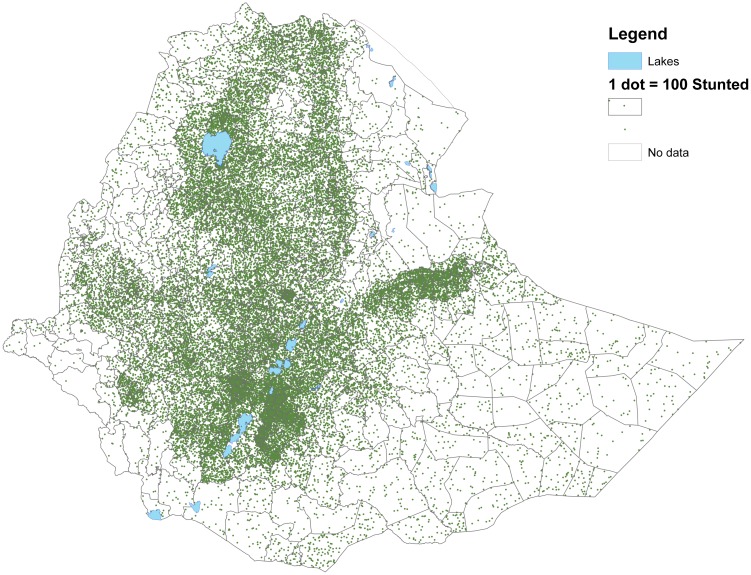
Number of children stunted in each woreda. The figure shows the number of stunted children. Each dot represent 100 stunted children.

**Fig 7:**
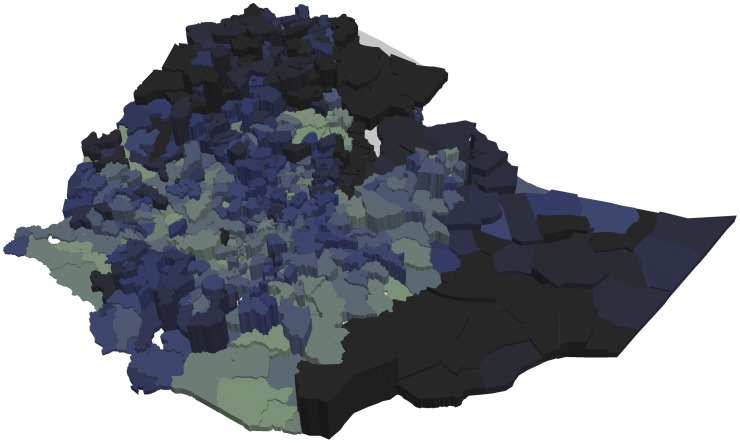
Share and number of underweight children in each woreda. The figure shows the number of underweight children by the height of the column and the share of children underweight by the color of each woreda. The color scale is the same as in Fig 3.

**Fig 8:**
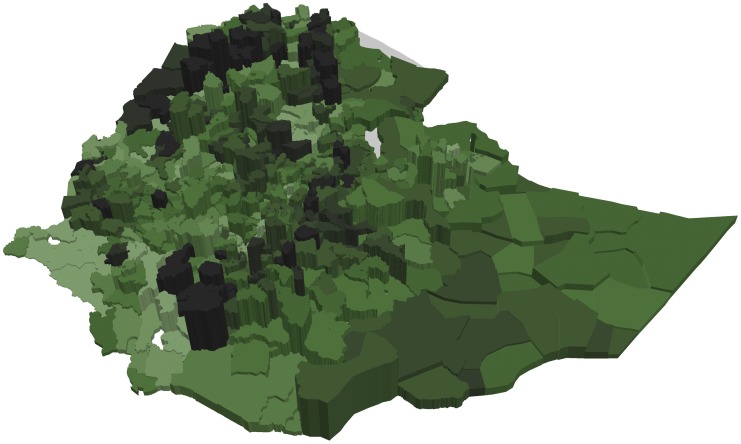
Share and number of stunted children in each woreda. The figure shows the number of stunted children by the height of the column and the share of children stunted by the color of each woreda. The color scale is the same as in Fig 4.

Figs 3 and 4 show that woredas further away from the center of the country tend to have higher share of underweight and stunted children than woredas closer to the center (with some notable variation). This is in contrast to Figs 5 and 6 showing the number of undernourished children (each dot represents 100 undernourished). Figs 5 and 6 show that in many areas most stunted or underweight children live close to the center of the country. This combined pattern is illustrated jointly in Figs 7 and 8, with height of columns showing the number of underweight and stunted children, while the colors illustrate the share of underweight and stunted children. This shows how some areas, like the dry areas in Somali (to the southeast) have a relatively high share of undernourished children but also a small population, and, therefore a low absolute number of undernourished children. Other areas, like the center of country, have a large population with a low share of undernourished, captured through tall columns and light colors in Figs 7 and 8. A few areas, like the north west, seem to have both a high share of undernourished and a large absolute number of undernourished.

**Fig 9:**
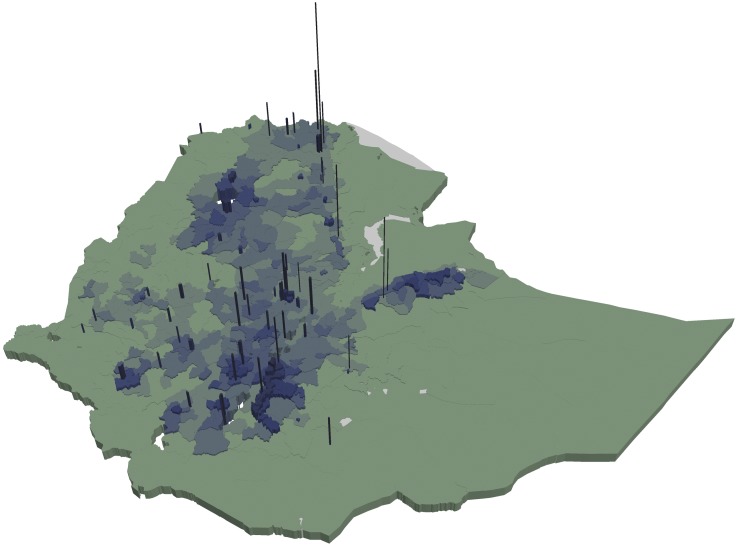
Concentration of underweight children (number of underweight children per km2). The figure shows the number of underweight children per km2 in each woreda.

**Fig 10:**
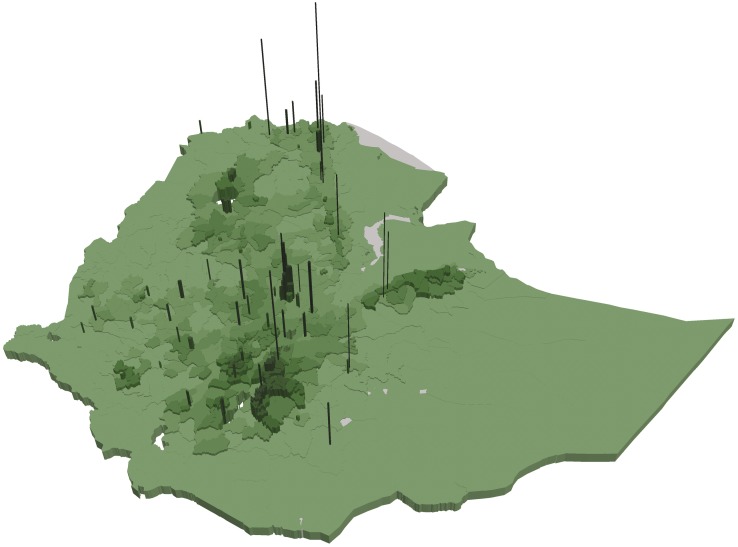
Concentration of stunted children (number of stunted children per km2). The figure shows the number of stunted children per km2 in each woreda.

Further, more urban areas, though having a relative low share of undernourished children[8], have a large population, and a large population that is spatially very concentrated. This concentration is illustrated by Figs 9 and 10 where the height of columns show that the number of children per km2 is very high in some locations.

**Fig 11:**
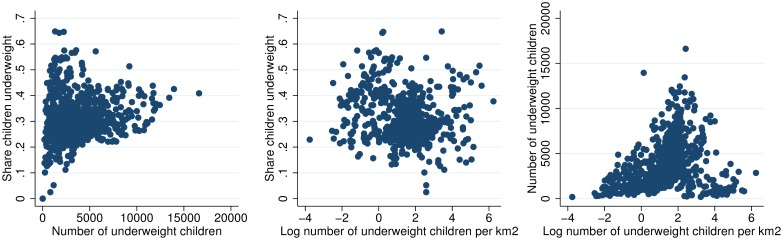
Spatial correlation between share, number and concentration of underweight children.

**Fig 12:**
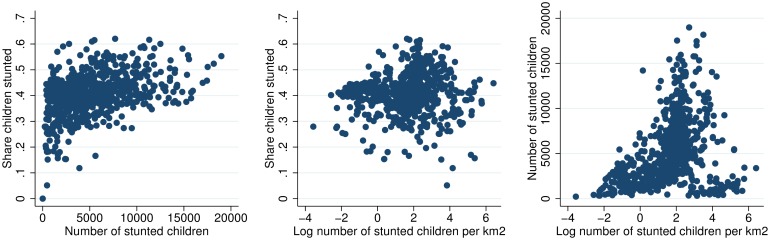
Spatial correlation between share, number and concentration of stunted children.

Figs 3 to 10 illustrate that different statistics (share, number, and number of children per km2) all support an understanding of where the challenges are largest. Some woredas are among the worst by one statistic, but among the better off by another. That one statistic is insufficient for understanding the spatial distribution of underweight and stunting is further illustrated in Figs 11 and 12 that show the spatial correlation among the different woreda statistics (share, number and number per km2) for underweight and stunting, respectively. Figs 11 and 12 show that for both underweight and stunting, there is very limited correlation between these statistics (share, number and number per km2).

**Fig 13:**
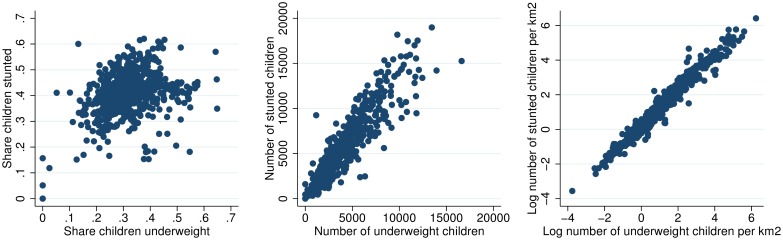
Location of underweight and stunted children.

The spatial correlation between stunting and underweight is also of interest. Fig 13 shows that the correlation between share of children stunted and underweight in each woreda is noisy (image to the left). However, the correlation between number of children underweight and stunted (image in the center), and number of underweight and stunted per km2 (images in the center and to the right), is high. The pattern reflect that the variation in prevalence of stunting and underweight observed in the left panel is not sufficient to dominate the spatial concentration of the population of children. For instance, two small woredas of 1000 children each, could have a prevalence of 20 and 30, resulting in 200 and 300 undernourished children in each. Other two larger woredas of say 10000 children in each, with same prevalence would have 2000 and 3000 undernourished children. In this case, though some woreads have same prevalence, the two larger woredas with very different prevalence would have a higher correlation, as observed in the two left images in Fig 13. The pattern can also be observed in the maps, where Figs 9 and 10 look similar, while Figs 3 and 4 are less similar.

## Concluding remarks

This paper contributes the first estimates of child undernutrition for all woredas in Ethiopia. Hence, the paper contributes to the rich literature on *who* is undernourished with the first estimates of *where* the undernourished resides. It further shows that SAE models of undernutrition have as much or more predictive power than common observed correlates of undernutrition. As such, the estimates can be used to address the concerns highlighted by Rajkumar et al.[4] in the introduction “*Ethiopia’s malnutrition rate could probably be much reduced by shifting some of the programs from the woredas with a high concentration of major programs into woredas with high malnutrition*”. The results provide the data to proof or disproof Rajkumar et al.’s claim and assess whether current efforts are as effective as they can be, and where there might be need for new or additional efforts. The results also show that woredas with “high malnutrition”, as referenced above, depends on the statistic utilized. Woredas with a high share of undernourished are often not the same as those with many undernourished, or a high concentration of undernourished (number of undernourished per km2). These aspects could inform the type of interventions used in different locations. The spatial correlation of underweight and stunting, could also be informative for planning purposes, as the number and concentration of undernourished children is high, while there is much less correlation between the share of undernourished children.

## Supporting information

S1 Table Mean in survey and census of variables in prediction models. (DOCX)

S2 Table Regional prediction models for stunting and underweight. (DOCX)
