# Supplementary material for: Small area estimation of child undernutrition in Ethiopian woredas
Source: PLoS One. 2017 Apr 14;12(4):e0175445. doi: 10.1371/journal.pone.0175445 (PMC5391934; doi:10.1371/journal.pone.0175445)
Supplement: S1 Table — (DOCX) [file pone.0175445.s001.docx]

**S1 Table. Mean in survey and census of variables in prediction models**

|  | Tigray | | Afar | | Amhara | | Oromiya | | Somali | | B. Gumuz | | SNNP | | Gambela | |
| --- | --- | --- | --- | --- | --- | --- | --- | --- | --- | --- | --- | --- | --- | --- | --- | --- |
|  | surv | cens | surv | cens | surv | cens | surv | cens | surv | cens | surv | cens | surv | cens | surv | cens |
| Child age = 0 | **0.17** | **0.19** | 0.19 | 0.10 | 0.18 | 0.18 | **0.18** | **0.17** | 0.16 | 0.07 | 0.19 | 0.17 | 0.17 | 0.16 | 0.16 | 0.15 |
| Child age = 1 |  |  | **0.15** | **0.15** | **0.18** | **0.19** | **0.16** | **0.19** | **0.18** | **0.14** | **0.20** | **0.19** | **0.16** | **0.18** | **0.15** | **0.18** |
| Child age = 2 |  |  | **0.24** | **0.23** | **0.19** | **0.20** | **0.19** | **0.22** | **0.22** | **0.26** | **0.21** | **0.22** | **0.19** | **0.20** | **0.18** | **0.21** |
| Child age = 3 | **0.21** | **0.21** | **0.22** | **0.22** | **0.23** | **0.20** |  |  | **0.22** | **0.19** | **0.19** | **0.19** | **0.24** | **0.22** | **0.25** | **0.23** |
| Child age = 4 | **0.22** | **0.21** | **0.21** | **0.30** | **0.22** | **0.22** | **0.22** | **0.24** | **0.22** | **0.34** | **0.21** | **0.22** | **0.23** | **0.24** | **0.25** | **0.23** |
| Gender: female | **1.46** | **1.50** |  |  | 1.50 | 1.50 |  |  |  |  |  |  |  |  |  |  |
| No. of HH members = 4 |  |  |  |  |  |  |  |  |  |  |  |  |  |  | 0.20 | 0.19 |
| No. of Small children = 1 | 0.35 | 0.39 |  |  |  |  |  |  |  |  |  |  |  |  |  |  |
| No. of Small children = 2 |  |  |  |  |  |  | 0.47 | 0.47 |  |  |  |  |  |  |  |  |
| No. of young = 1 |  |  | **0.09** | **0.08** |  |  |  |  | **0.17** | **0.20** |  |  |  |  |  |  |
| No. of young = 3 |  |  |  |  | **0.24** | **0.26** |  |  |  |  |  |  |  |  |  |  |
| No. of young = 4 |  |  | 0.22 | 0.23 |  |  |  |  |  |  |  |  |  |  | 0.21 | 0.22 |
| No. of adults = 3 |  |  |  |  | **0.18** | **0.20** |  |  |  |  |  |  |  |  |  |  |
| No. of adults = 4 |  |  |  |  |  |  |  |  |  |  |  |  |  |  | 0.09 | 0.09 |
| Male HH head |  |  |  |  |  |  |  |  |  |  |  |  | **0.89** | **0.85** |  |  |
| Age of HH head |  |  |  |  |  |  |  |  |  |  |  |  |  |  | **37.9** | **36.0** |
| Spouse in HH |  |  |  |  | 0.88 | 0.89 |  |  |  |  |  |  |  |  |  |  |
| Rural |  |  |  |  |  |  |  |  | **0.62** | **0.88** |  |  |  |  | **0.80** | **0.77** |
| Water source: Public tap |  |  |  |  |  |  | **0.15** | **0.16** |  |  |  |  |  |  |  |  |
| Roof material: Thatch |  |  |  |  |  |  |  |  |  |  |  |  | 0.67 | 0.67 | **0.64** | **0.68** |

Notes: Values in **bold** are for variables used in the height-for-age- model; values that are underlined are for variables used in the weight-for-age model.
