# Supplementary material for: Small area estimation of child undernutrition in Ethiopian woredas
Source: PLoS One. 2017 Apr 14;12(4):e0175445. doi: 10.1371/journal.pone.0175445 (PMC5391934; doi:10.1371/journal.pone.0175445)
Supplement: S2 Table — (DOCX) [file pone.0175445.s002.docx]

**S2 Table. Regional prediction models for stunting and underweight**

| **Tigray height-for-age** |  |  |  |
| --- | --- | --- | --- |
|  | Coef. | SE | P |
| Child age 0 | 1.810 | 0.19 | 0.000 |
| Child age 4 | 0.296 | 0.17 | 0.077 |
| EA avg. age of HH head | -0.070 | 0.02 | 0.000 |
| EA avg. Floor material: mud | 1.460 | 0.55 | 0.008 |
| EA avg. Male HH head | -2.312 | 0.67 | 0.001 |
| EA avg. Number of rooms: 1 | 0.952 | 0.29 | 0.001 |
| EA avg. Self employed | 0.379 | 0.23 | 0.095 |
| Gender: female | 0.230 | 0.14 | 0.092 |
| Zone = 2 | 0.790 | 0.18 | 0.000 |
| Constant | 0.235 | 0.80 | 0.768 |
| Obs. in census | 107,107 |  |  |
| Obs. in survey | 435 |  |  |
| R^2 | 0.261 |  |  |
| Adj. R^2 | 0.245 |  |  |

| **Afar height-for-age** |  |  |  |
| --- | --- | --- | --- |
|  | Coef. | SE | P |
| Child age 1 | -1.75 | 0.38 | 0.00 |
| Child age 2 | -1.75 | 0.34 | 0.00 |
| Child age 3 | -1.66 | 0.35 | 0.00 |
| Child age 4 | -1.60 | 0.34 | 0.00 |
| EA avg. Light source: Firewood | 1.15 | 0.38 | 0.00 |
| EA avg. Orphans | -7.47 | 1.87 | 0.00 |
| No small children in HH= 1 | 2.43 | 1.15 | 0.03 |
| Constant | 0.15 | 0.29 | 0.61 |
| Obs. in census | 26,089 |  |  |
| Obs. in survey | 565 |  |  |
| R^2 | 0.133 |  |  |
| Adj. R^2 | 0.122 |  |  |

| **Amhara height-for-age** |  |  |  |
| --- | --- | --- | --- |
|  | Coef. | SE | P |
| Child age 1 | -1.235 | 0.20 | 0.000 |
| Child age 2 | -1.252 | 0.20 | 0.000 |
| Child age 3 | -1.127 | 0.19 | 0.000 |
| Child age 4 | -1.217 | 0.19 | 0.000 |
| EA avg. Water source: public tap | 1.098 | 0.4 | 0.006 |
| EA avg. Water source: unprotected spring | 1.218 | 0.26 | 0.000 |
| EA avg. Children age 5-10 enrolled in school | -3.289 | 0.86 | 0.000 |
| EA avg. House age less than 10 years | -1.992 | 0.61 | 0.001 |
| Number of adults: 3 | 0.275 | 0.15 | 0.067 |
| Number of Young: 3 | 0.223 | 0.14 | 0.107 |
| Zone = 2 | 0.822 | 0.26 | 0.002 |
| Zone = 3 | 0.491 | 0.23 | 0.034 |
| Zone = 4 | 1.207 | 0.27 | 0.000 |
| Zone = 5 | 0.545 | 0.38 | 0.148 |
| Zone = 6 | 0.828 | 0.23 | 0.000 |
| Zone = 7 | 0.929 | 0.25 | 0.000 |
| Zone = 8 | 1.492 | 0.77 | 0.052 |
| Zone = 9 | 0.851 | 0.28 | 0.002 |
| Zone = 10 | 0.844 | 0.32 | 0.009 |
| Constant | -0.994 | 0.30 | 0.001 |
| Obs. in census | 419,466 |  |  |
| Obs. in survey | 518 |  |  |
| R^2 | 0.203 |  |  |
| Adj. R^2 | 0.173 |  |  |

| **Oromiya height-for-age** |  |  |  |
| --- | --- | --- | --- |
|  | Coef. | SE | P |
| Child age 0 | 2.66 | 0.19 | 0.00 |
| Child age 1 | 0.83 | 0.20 | 0.00 |
| Child age 4 | 0.61 | 0.17 | 0.00 |
| Water source: public tap | -0.63 | 0.22 | 0.00 |
| EA avg. Cooking fuel is dung | -0.67 | 0.18 | 0.00 |
| EA avg. Number of disabled | -3.45 | 1.71 | 0.04 |
| EA avg. House age less than 5 years | -1.23 | 0.34 | 0.00 |
| EA avg. Houses with livestock | 1.04 | 0.48 | 0.03 |
| EA avg. Share has employment | 4.27 | 1.28 | 0.00 |
| Constant | -2.35 | 0.47 | 0.00 |
| Obs. in census | 734,674 |  |  |
| Obs. in survey | 635 |  |  |
| R^2 | 0.267 |  |  |
| Adj. R^2 | 0.256 |  |  |

| **Somali height-for-age** |  |  |  |
| --- | --- | --- | --- |
|  | Coef. | SE | P |
| Child age 1 | -2.04 | 0.35 | 0.00 |
| Child age 2 | -2.15 | 0.34 | 0.00 |
| Child age 3 | -2.46 | 0.34 | 0.00 |
| Child age 4 | -2.38 | 0.34 | 0.00 |
| EA avg. Water source: river | -0.63 | 0.26 | 0.01 |
| EA avg. Water source: unprotected spring | -0.34 | 0.24 | 0.16 |
| Number young in HH:3 | -0.53 | 0.27 | 0.05 |
| Rural | -0.32 | 0.23 | 0.16 |
| Constant | 1.33 | 0.35 | 0.00 |
| Obs. in census | 75,249 |  |  |
| Obs. in survey | 591 |  |  |
| R^2 | 0.169 |  |  |
| Adj. R^2 | 0.158 |  |  |

| **B. Gumuz height-for-age** |  |  |  |
| --- | --- | --- | --- |
|  | Coef | SE | P |
| Child age 1 | -0.86 | 0.38 | 0.02 |
| Child age 2 | -1.37 | 0.36 | 0.00 |
| Child age 3 | -1.22 | 0.37 | 0.00 |
| Child age 4 | -1.45 | 0.36 | 0.00 |
| EA avg. Young HH member | -7.42 | 1.42 | 0.00 |
| EA avg. House age less than 10 years | 4.94 | 0.84 | 0.00 |
| EA avg. HH has livestock | 2.18 | 0.40 | 0.00 |
| EA avg. Number of rooms: 1 | 1.37 | 0.36 | 0.00 |
| EA avg. Share employed | 1.61 | 0.77 | 0.04 |
| Constant | 1.95 | 1.32 | 0.14 |
| Obs. in census | 21,542 |  |  |
| Obs. in survey | 425 |  |  |
| R^2 | 0.186 |  |  |
| Adj. R^2 | 0.168 |  |  |

| **SNNP height-for-age** |  |  |  |
| --- | --- | --- | --- |
|  | Coef. | SE | P |
| Child age 1 | -1.594 | 0.26 | 0.000 |
| Child age 2 | -1.729 | 0.25 | 0.000 |
| Child age 3 | -2.142 | 0.23 | 0.000 |
| Child age 4 | -2.030 | 0.23 | 0.000 |
| EA avg. House age less than 15 years | 2.481 | 0.68 | 0.000 |
| EA avg. HH has radio | 1.201 | 0.39 | 0.002 |
| Male HH head | -0.496 | 0.24 | 0.038 |
| Zone = 2 | 0.701 | 0.32 | 0.029 |
| Zone = 4 | 0.229 | 0.21 | 0.265 |
| Zone = 6 | 0.459 | 0.32 | 0.153 |
| Zone = 7 | 1.276 | 0.47 | 0.007 |
| Zone = 9 | 1.236 | 0.34 | 0.000 |
| Zone = 11 | 1.091 | 0.29 | 0.000 |
| Zone = 17 | 0.866 | 0.47 | 0.066 |
| Zone = 20 | 0.928 | 0.38 | 0.014 |
| Zone = 21 | 1.710 | 0.62 | 0.006 |
| Constant | -0.676 | 0.36 | 0.061 |
| Obs. in census | 402,868 |  |  |
| Obs. in survey | 673 |  |  |
| R^2 | 0.213 |  |  |
| Adj. R^2 | 0.192 |  |  |

| **Gambela height-for-age** |  |  |  |
| --- | --- | --- | --- |
|  | Coef. | SE | P |
| Child age 1 | -0.838 | 0.39 | 0.034 |
| Child age 2 | -1.506 | 0.37 | 0.000 |
| Child age 3 | -1.718 | 0.34 | 0.000 |
| Child age 4 | -1.106 | 0.34 | 0.001 |
| Age of HH head | 0.020 | 0.01 | 0.019 |
| EA avg. Government employed | 2.013 | 0.57 | 0.001 |
| Rural | -0.715 | 0.37 | 0.052 |
| Roof material: Thatch | 0.732 | 0.32 | 0.023 |
| Constant | -0.617 | 0.53 | 0.250 |
| Obs. in census | 7,148 |  |  |
| Obs. in survey | 403 |  |  |
| R^2 | 0.198 |  |  |
| Adj. R^2 | 0.178 |  |  |

| **Tigray weight-for-age** |  |  |  |
| --- | --- | --- | --- |
|  | Coef.t | SE | P |
| Child age 0 | 0.42 | 0.14 | 3.02 |
| EA avg. Ceiling quality | 1.61 | 0.56 | 2.88 |
| EA avg.Heating kerosene | -2.13 | 0.46 | -4.61 |
| EA avg. First grade | -7.48 | 2.02 | -3.71 |
| EA avg. Male head | -1.23 | 0.40 | -3.09 |
| EA avg. Number of rooms: 2 | -0.64 | 0.30 | -2.15 |
| EA avg. Share adults employed | -0.85 | 0.35 | -2.43 |
| Constant | 0.19 | 0.36 | 0.54 |
| Obs. in census | 107,107 |  |  |
| Obs. in survey | 445 |  |  |
| R^2 | 0.127 |  |  |
| Adj. R^2 | 0.112 |  |  |

| **Afar weight-for-age** |  |  |  |
| --- | --- | --- | --- |
|  | Coefficient | SE | P |
| Child age 0 | -0.38 | 0.21 | -1.79 |
| Child age 2 | -0.69 | 0.19 | -3.59 |
| Child age 3 | -0.54 | 0.20 | -2.73 |
| EA avg. Garbage burnt | -0.74 | 0.22 | -3.41 |
| EA avg. First grade | 4.05 | 2.28 | 1.77 |
| EA avg. House age less than 10 years | -1.64 | 0.68 | -2.40 |
| EA avg. Number of dead children | 1.65 | 0.38 | 4.29 |
| Kebele avg. Public tab | -1.19 | 0.39 | -3.07 |
| Number of young: 4 | -0.56 | 0.18 | -3.12 |
| Constant | -1.36 | 0.18 | -7.69 |
| Obs. in census | 26,089 |  |  |
| Obs. in survey | 608 |  |  |
| R^2 | 0.117 |  |  |
| Adj. R^2 | 0.104 |  |  |

| **Amhara weight-for-age** |  |  |  |
| --- | --- | --- | --- |
|  | Coef. | SE | P |
| Child age 0 | 0.618 | 0.12 | 0.000 |
| EA avg. HH has Ceiling | 3.607 | 1.56 | 0.021 |
| EA avg. Water source: Unprotected spring | 0.621 | 0.16 | 0.000 |
| EA avg. Children age 5-10 enrolled in school | -1.839 | 0.53 | 0.001 |
| EA avg. Light source: Electricity | -2.197 | 0.74 | 0.003 |
| EA avg. Number of rooms | 0.450 | 0.10 | 0.000 |
| EA avg. Religion: Muslim | 0.895 | 0.16 | 0.000 |
| Gender: female | -0.157 | 0.09 | 0.078 |
| Number of Young: 3 | 0.329 | 0.10 | 0.002 |
| Spouse in HH | -0.352 | 0.16 | 0.025 |
| Constant | -2.127 | 0.22 | 0.000 |
| Obs. in census | 419,466 |  |  |
| Obs. in survey | 523 |  |  |
| R^2 | 0.157 |  |  |
| Adj. R^2 | 0.140 |  |  |

| **Oromiya weight-for-age** |  |  |  |
| --- | --- | --- | --- |
|  | Coef. | SE | P |
| Child age 0 | 1.063 | 0.14 | 0.000 |
| EA avg. Cooking fuel: Dung | -0.372 | 0.14 | 0.006 |
| EA avg. Cooking fuel: fire | -0.555 | 0.26 | 0.032 |
| EA avg. Floor material: Mud | -3.424 | 0.78 | 0.000 |
| EA avg. House age less than 20 years | 0.881 | 0.38 | 0.019 |
| EA avg. Livestock lives in HH | 2.073 | 0.42 | 0.000 |
| EA avg. Orphan HH member | 3.958 | 1.76 | 0.025 |
| Number of small children: 2 | -0.232 | 0.11 | 0.029 |
| Constant | 0.695 | 0.59 | 0.237 |
| Obs. in census | 734,674 |  |  |
| Obs. in survey | 656 |  |  |
| R^2 | 0.135 |  |  |
| Adj. R^2 | 0.124 |  |  |

| **Somali weight-for-age** |  |  |  |
| --- | --- | --- | --- |
|  | Coef. | SE | P |
| Child age 0 | 1.682 | 0.21 | 0.000 |
| EA avg. Cooking fuel: Other | -0.981 | 0.36 | 0.007 |
| EA avg. Light source: Lantern | 0.534 | 0.24 | 0.025 |
| EA avg. Roof material: Thatch | -0.900 | 0.25 | 0.000 |
| EA avg. Wall material: Wood or Mud | -1.256 | 0.27 | 0.000 |
| Constant | -0.759 | 0.25 | 0.003 |
| Obs. in census | 75,249 |  |  |
| Obs. in survey | 591 |  |  |
| R^2 | 0.173 |  |  |
| Adj. R^2 | 0.166 |  |  |

| **B. Gumuz weight-for-age** |  |  |  |
| --- | --- | --- | --- |
|  | Coef. | SE | P |
| Child age 0 | 0.98 | 0.22 | 0.00 |
| EA avg. Migrant HH | -6.66 | 2.45 | 0.01 |
| EA avg. Number of orphants | -13.09 | 2.44 | 0.00 |
| EA avg. Enrollment third grade | -6.23 | 1.65 | 0.00 |
| EA avg: Has employment | 10.51 | 2.23 | 0.00 |
| EA avg. Roof material: Thatch | 1.77 | 0.34 | 0.00 |
| EA avg. Waste disposal burned | -2.03 | 0.38 | 0.00 |
| EA avg. Wall material: wood and mud | 1.52 | 0.24 | 0.00 |
| Constant | -2.24 | 0.41 | 0.00 |
| Obs. in census | 21,542 |  |  |
| Obs. in survey | 437 |  |  |
| R^2 | 0.253 |  |  |
| Adj. R^2 | 0.239 |  |  |

| **SNNP weight-for-age** |  |  |  |
| --- | --- | --- | --- |
|  | Coef. | SE | P |
| Child age 0 | 0.688 | 0.15 | 0.000 |
| Child age 4 | -0.437 | 0.13 | 0.001 |
| EA avg. House age less than 15 years | 3.359 | 0.60 | 0.000 |
| EA avg. House age less than 20 years | -3.556 | 0.91 | 0.000 |
| EA avg. Kitchen outside | -1.201 | 0.35 | 0.001 |
| EA avg. Light source: firewood | 5.156 | 0.98 | 0.000 |
| EA avg. Male head of HH | -1.179 | 0.51 | 0.021 |
| EA avg. Number of rooms: 2 | -0.456 | 0.21 | 0.028 |
| EA avg. House ownership | 0.665 | 0.29 | 0.022 |
| EA avg. Education level: Third grade | 2.493 | 0.76 | 0.001 |
| Roof material: Thatch | -0.254 | 0.12 | 0.031 |
| Constant | -0.927 | 0.50 | 0.066 |
| Obs. in census | 402,868 |  |  |
| Obs. in survey | 699 |  |  |
| R^2 | 0.168 |  |  |
| Adj. R^2 | 0.154 |  |  |

| **Gambela weight-for-age** |  |  |  |
| --- | --- | --- | --- |
|  | Coef. | SE | P |
| Child age 0 | 0.373 | 0.21 | 0.074 |
| EA avg. Water source: unprotected spring | 0.533 | 0.32 | 0.098 |
| EA avg. Government employed | 1.248 | 0.38 | 0.001 |
| Number of HH members: 4 | -0.688 | 0.26 | 0.008 |
| Number of adults: 4 | -0.504 | 0.25 | 0.046 |
| Number of young: 4 | -0.330 | 0.19 | 0.078 |
| Rural | -0.366 | 0.23 | 0.117 |
| Constant | -0.727 | 0.23 | 0.002 |
| Obs. in census | 7,148 |  |  |
| Obs. in survey | 401 |  |  |
| R^2 | 0.112 |  |  |
| Adj. R^2 | 0.096 |  |  |
